# Tissue culture requirements of the evergreen broad-leaved plant *Illicium henryi* D

**DOI:** 10.3389/fpls.2025.1581069

**Published:** 2025-09-10

**Authors:** Panpan Li, Ying Yang, Songlin Jiang, Kexin Chen, Ninghan Xue, Xingran Ji, Xiaoge Wang, Wenli Ji

**Affiliations:** ^1^ College of Landscape Architecture Art, Northwest A&F University, Xianyang, Shaanxi, China; ^2^ College of Landscape Architecture, Northeast Forestry University, Haerbin, Heilongjiang, China; ^3^ Faculty of Ecological and Environmental Engineering, Yangling Vocational & Technology College, Yangling, Shaanxi, China

**Keywords:** callus, evergreen broad-leaved plant, *Illicium henryi*, *In vitro* tissue culture, plant growth regulator

## Abstract

*Illicium henryi* Diels is a rare wild plant species with limited natural distribution, ecological vulnerability, and challenging cultivation requirements, yet little progress has been made in its introduction and propagation techniques. To address this gap, this study aimed to establish an efficient protocol for its aseptic plantlet production using tissue culture technology. Semi-lignified stem segments with buds, young leaves, sprouting buds, and young shoots bearing buds from the current year were selected as explants, and the effects of different sterilization methods and explant types on disinfection efficiency were systematically evaluated. Based on these findings, suitable basal media were identified for bud induction and leaf-derived callus induction, and the roles of plant growth regulators including 6-BA, NAA, and IBA were further assessed at different stages of bud induction, callus formation, and proliferation. The optimized conditions enabled the successful in vitro regeneration of *I. henryi* plantlets, with both bud-bearing stem segments and leaf-derived callus showing stable regeneration potential. These results provide a practical method for the artificial propagation of *I. henryi* in introduced habitats and establish a reliable regeneration system, thereby contributing to its conservation, sustainable utilization, and future tissue culture research in *Illicium* and related species.

## Introduction

*Illicium henryi* Diels, belonging to the family Schisandraceae, is an evergreen broad-leaved shrub or small tree. It is characterized by alternate leathery leaves and red, pendulous flowers resembling inverted lotus blossoms, which are borne in axillary or sub-terminal positions. With its dense foliage and year-round greenery, the species holds significant ornamental value in landscape applications (Liang et al., 2012). In recent years, studies have shown that extracts of *I. henryi*, which has traditionally been used in folk medicine for its anti-inflammatory and analgesic effects (Min et al., 2000), also promotes angiogenesis in osteoporotic fracture models in rats (Shi et al., 2023) and exhibits antiviral and cytotoxic activities (Yong et al., 2023), highlighting its pharmacological potential. Despite its promising prospects in both landscape and medicinal applications, the biological resources of this species remain underutilized.

The natural populations of *I. henryi* are sparsely and discontinuously distributed in the wild, and its cultivation remains limited (Liang et al., 2012). Moreover, its propagation efficiency is notably low. Previous studies have reported that softwood cuttings treated with plant growth regulators (PGRs) such as α-naphthaleneacetic acid (NAA) and indole-3-butyric acid (IBA) exhibit a rooting rate of less than 20%, while seed propagation results in a germination rate of only 62%, indicating that conventional methods fail to meet the requirements for large-scale cultivation and introduction (Shi et al., 2012).

Tissue culture technology offers an efficient approach for the propagation and germplasm conservation of species that are difficult to propagate by conventional means (Bertsouklis et al., 2024; Shivani et al., 2024). However, systematic studies on the tissue culture of *I. henryi* are still lacking. Previous research has primarily focused on related species within the genus, addressing aspects such as explant sterilization protocols, optimal culture medium formulations, and conditions for adventitious bud induction. For instance, in *Illicium lanceolatum* cv. Haierlian, treating sprouting buds with 0.1% HgCl_2_ for 6 minutes effectively controlled contamination (Fang et al., 2012); in *Schisandra chinensis*, callus was successfully induced from tender stems and later developed into adventitious buds (Sun et al., 2016); and in *Illicium difengpi*, optimal basal media for both initial and subculture stages were established (Li et al., 2015), providing useful references for this study.

In plant tissue culture, regeneration typically occurs via two main pathways: direct organogenesis, where adventitious shoots or roots develop directly from the pre-existing meristematic tissues of the explants, and indirect organogenesis, which involves callus formation followed by organ differentiation (Hesami et al., 2019; Khan et al., 2021; Long et al., 2022). Due to the highly differentiated tissue structure of woody plants, inducing adventitious buds from callus is often inefficient. Therefore, stem segments with buds are commonly used as explants for rapid multiplication via direct organogenesis (Anushi et al., 2023; Dias et al., 2016).

Given the unclarified regenerative potential of callus tissue in *I. henryi*, this study selected various explant types including current-year semi-lignified bud-bearing stem segments, sprouting buds, young leaves, and tender shoots with buds. A systematic comparison was conducted to evaluate the effects of different combinations of PGRs on both direct and indirect organogenesis. Through sterilization optimization, explant screening, and induction experiments, this study aimed to identify optimal medium formulations for bud induction, callus induction, and subsequent proliferation. These findings will contribute to establishing an efficient propagation system for *I. henryi* in introduced regions and provide technical support for its germplasm utilization and conservation.

## Materials and methods

### Description of the experimental site

The sampling site was the Expo Garden of Northwest A&F University (108°4′21.076′′E, 34°15′57.593′′N), situated in the central Guanzhong Plain of Shaanxi Province, China. The region experiences a continental monsoon-influenced semi-humid climate characterized by four distinct seasons. Precipitation predominantly occurs between July and September, with an annual average temperature of 12.9°C and a frost-free period of 211 days.

### Plant materials

**Figure 1:**
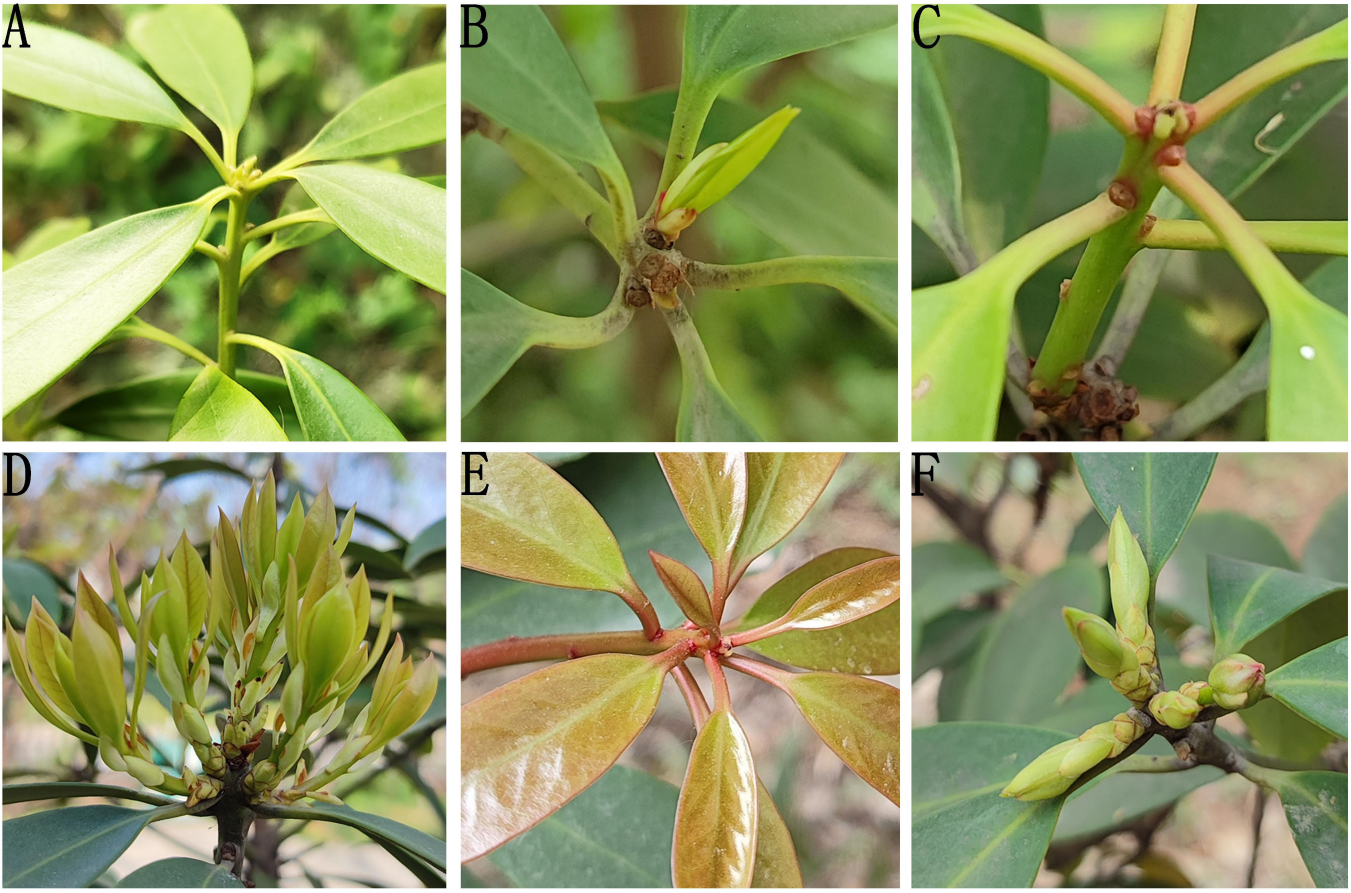
The four explant types **(A)** current-year semi-Lignified stem segments with buds in summer; **(B)** young leaves and sprouting buds in summer; **(C)** young stem with buds in summer; **(D, E)** young leaves in spring; **(F)** sprouting buds in spring.

*I. henryi* plants cultivated in the Expo Garden of Northwest A&F University were used as the plant material. Samples were collected at irregular intervals from July 2023 to May 2024. On each sampling day, vigorously growing, disease- and pest-free tissues were selected, consisting of four types of explants: bud-bearing stem segments (with apical or axillary buds), emerged buds, tender bud-bearing stem segments, and young leaves, which were used as explants (**Figure 1**). The explant materials were collected at the following times: in the summer of 2023 (July and August), semi-lignified stem segments with buds (current-year materials with slight lignification), sprouting buds, young stem segments with buds, and leaves were collected; in the spring of 2024 (March, April, and May), sprouting buds, young stem segments with buds, and leaves were collected.

### Culture conditions and *in vitro* culture experiments

The tissue culture system and experimental procedures used in this study are summarized in **Table 1**. A two-factor, three-level orthogonal experimental design was employed to optimize the basal medium type and plant growth regulator combinations, with the aim of establishing an efficient regeneration system for aseptic plantlets of *I. henryi*. All cultures were maintained under controlled conditions at 23 ± 2 °C with a photoperiod of 12 h/day and a light intensity of 1000–2000 lx. The culture media were supplemented with 8 g/L agar and 30 g/L sucrose, and the pH was adjusted to 5.8–6.2 prior to autoclaving.

**Table 1:** Summary of experimental designs and key parameters for Illicium henryi in vitro culture across developmental stages.

Culture stage		Explant type	Sterilization treatment	Basal medium	Plant growth regulators (mg/L)	Culture period (days)	Data statistics	Observation content
Establishment of a sterilization system	Explant Pretreatment	Semi-lignified stem segments with buds	2% NaClO or 0.1% HgCl_2_	MS	6-BA 2.0 + NAA 0.5	10	Contamination and Browning Rate Statistics	Assessment of contamination and browning status following explant pretreatment
		Young leaves						
		Sprouting buds; Young bud-bearing stem segments						
Direct organogenesis pathway	Assessment of Culture media for Sprouting Bud Induction	Sprouting buds	–	MS, 0.5xMS, WPM	6-BA 2.0 + NAA 0.5	30	Shoot Multiplication Statistics	Evaluation of bud development on three media, including shoot induction number and days to initial sprouting
	Assessment of Plant Growth Regulators for the Induction of Budded Stem Segments	Bud-bearing stem segments	–	MS	6-BA (1.0, 1.5, 2.0) + NAA (0.05, 0.1, 0.5)	30	Axillary Bud Induction Rate	Monitoring of the growth performance of bud-bearing stem segments used as explants
	Evaluation of Culture media for Healthy plantlet Development	Sprouting buds	–	MS	6-BA (0.5, 1.0, 2.0) + NAA (0.1, 0.5)/ IBA (1.0, 2.0, 3.0)/KT 1.0	30	–	Documentation of shoot elongation occurrence, comparison of growth rates, and characterization of newly formed leaves
		Shoots induced from axillary buds						
	Root Induction	Well-developed plantlets	–	0.5xMS	NAA (0.1, 0.5, 1.0) + IBA (0.5, 1.0, 2.0)	30	–	Assessment of rooting performance
Indirect organogenesis pathway	callus Induction from Leaf Explants	Young leaves	–	MS, 0.5xMS, WPM	6-BA (1.0, 1.5, 2.0, 3.0) + NAA (0.1, 0.5)	30	callus Induction Rate and Browning Rate from Leaf Explants	Observation and evaluation of callus formation and browning at leaf explant cut edges
	callus Proliferation	Leaf-derived callus	–	MS, 0.5xMS	6-BA 2.0 + NAA (0.1, 0.5, 1.0)	30	–	Comparative analysis of callus growth status, texture changes, and proliferation capacity among treatment groups
	callus Differentiation	Proliferated callus	–	MS, 0.5xMS	6-BA (0.2, 0.5, 2.0), NAA (0.5, 1.0, 2.0), IBA (0.2, 0.5), KT 1.0	30	–	Observation of shoot primordia formation, including number and developmental stages

## Methods

### Selection of explant types and optimal sterilization methods

To optimize the sterilization protocol, the effects of two commonly used sterilizing agents, NaClO and HgCl_2_, were systematically compared, and sterilization procedures were developed for different types of explants to identify the most suitable sterilization conditions.

For semi-lignified stem segments with buds, old leaves were first removed, and the stems were cut into approximately 3 cm segments. After rinsing, the explants were immersed in 75% ethanol for 20 seconds, followed by thorough washing with sterile distilled water. Subsequently, sterilization was performed using either NaClO or HgCl_2_ (**Table 2**), with two drops of Tween-80 added as a surfactant in all treatments. After chemical treatment, explants were rinsed six times with sterile distilled water. The basal 0.3 cm of each segment was then trimmed to remove physiologically aged tissue before transferring the explants to the initial culture medium.

**Table 2:** Experimental design for sterilization of semi-lignified stem segments with buds.

Serial number	Sterilization reagents	Sterilization time (min)
A	2% NaClO	3, 5, 7, 10, 13, 15
B	0.1% HgCl_2_	12, 13, 14

For the young leaves, sprouting buds, and young stem segments with buds, the surfaces were first cleaned with dishwashing detergent and then rinsed under running water for 1 hour. The materials were then transferred to a sterile laminar flow hood for sterilization. The leaves and sprouting buds were treated with 0.1% HgCl_2_ for 4, 5.5, and 7 minutes, respectively, and the young stem segments with buds were treated for 7 minutes (Jafernik et al., 2020). Tween-80 was also added to the sterilization solution, after which the materials were rinsed six times with sterile water and excess water was removed. After treatment, the petioles and tips of the leaves were removed, and 1–2 incisions were made on the abaxial surface to sever the main veins. The sprouting buds were stripped of their bud scales and the basal lignified portion was cut off. The young stem segments were trimmed by excising the physiological tip by approximately 0.3 cm. During sterilization, slight agitation of the containers was used to enhance the contact between the sterilizing agents and the explants. All materials were inoculated onto the basal medium after treatment. The contamination and browning rates for each treatment group were recorded on the 10th day after inoculation to assess sterilization effectiveness.

### Direct organogenesis pathway

#### Assessment of basic culture media for the induction of sprouting buds

MS medium, characterized by a relatively high concentration of inorganic salts, is one of the most widely used basal media in plant tissue culture (Murashige and Skoog, 1962). The 0.5x MS medium is a modified version in which the concentrations of macronutrients, micronutrients, and iron salts are reduced by half, while the levels of vitamins and organic components remain unchanged. WPM medium, specifically formulated for woody plants, features a lower overall ionic strength and a reduced ratio of ammonium to nitrate nitrogen, better aligning with the physiological requirements of such species (Lloyd and McCown, 1981).

In this study, newly sprouting buds collected in the summer of 2023 were used as explants. Following surface sterilization, the explants were cultured on MS, 0.5x MS, and WPM basal media. All media were supplemented with 2.0 mg/L 6-BA and 0.5 mg/L NAA to promote the further growth and development of buds that had already formed meristematic structures, rather than to induce dedifferentiation and *de novo* regeneration (Fang, 2010). Three experimental groups were established, each with four explants and three replicates (**Table 3**). After 30 days of continuous culture, bud development was assessed based on the number of induced buds, the number of days to initial bud emergence, and the multiplication rate. These metrics were used to evaluate the effectiveness of different media in promoting bud induction.

**Table 3:** Experimental Design for Bud Induction on Different Basal Media.

Serial number	Basic culture base	Inoculated number	Inducible number
1	MS	4	21
1	MS	4	25
1	MS	4	22
2	0.5x MS	4	18
2	0.5x MS	4	16
2	0.5x MS	4	11
3	WPM	4	5
3	WPM	4	12
3	WPM	4	17

#### Assessment of induction media for tender stem segments with buds

As demonstrated in earlier experiments described in this study, MS medium was more suitable for the induction of newly sprouting buds. Based on this finding, MS medium was selected as the basal medium in the present study to further investigate how different concentration combinations of cytokinin 6-BA and auxin NAA regulate the growth potential of buds from semi-lignified stem segments with pre-existing meristematic structures. A two-factor, three-level orthogonal design was employed, with 6-BA concentrations set at 1.0, 1.5, and 2.0 mg/L, and NAA concentrations at 0.05, 0.1, and 0.5 mg/L, resulting in a total of nine treatment combinations. Each treatment involved 15 explants, with three replicates (**Table 4**).

**Table 4:** Experimental Design for Bud Induction on Different Basal Media.

Serial number	Hormones		Inoculated number
	6-BA(mg/L)	NAA(mg/L)	
1	1.00	0.05	45
2	1.00	0.1	45
3	1.00	0.5	45
4	1.50	0.05	45
5	1.50	0.1	45
6	1.50	0.5	45
7	2.00	0.05	45
8	2.00	0.1	45
9	2.00	0.5	45

The explants were cultured for 30 days, during which their growth performance was monitored. Axillary bud induction rates were recorded to evaluate the effects of different plant growth regulator combinations on bud induction.

#### Assessment of culture media for healthy plantlet development

Sprouted adventitious buds successfully induced in the primary culture were selected as materials for plantlet development. After separation from the mother plant, they were inoculated onto media with nine different combinations of plant growth regulators (**Table 5**) to evaluate their growth and development. Ten buds were inoculated for each treatment group, with three replications. The cultures were maintained under the same controlled environmental conditions (e.g., temperature, light, and humidity) throughout the entire culture period, regardless of the different growth regulator treatments applied, and regular observations were made to monitor the growth dynamics of the buds.

**Table 5:** Hormone combination table of plantlet strengthening media.

Serial number	Hormone combinations for culture media
1	MS+6-BA 1.0mg/L+NAA 0.1mg/L+KT 1.0 mg/L
2	MS+6-BA 2.0mg/L+NAA 0.5mg/L+KT 1.0 mg/L
3	MS+6-BA 1.0mg/L+NAA 0.1mg/L+IBA 1.0 mg/L
4	MS+6-BA 2.0mg/L+NAA 0.5mg/L+IBA 1.0 mg/L
5	MS+6-BA 2.0mg/L+NAA 0.5mg/L
6	MS+6-BA 1.0mg/L+IBA 1.0mg/L
7	MS+6-BA 0.5mg/L+IBA 2.0mg/L
8	MS+6-BA 0.5mg/L+IBA 3.0mg/L
9	MS

The growth of the buds was evaluated by comparing the changes in bud height and leaf development before and after culture. Key observations focused on whether the buds exhibited significant elongation, differences in growth rate, and the number and morphological characteristics of new leaves. These parameters were used to comprehensively assess the promotive effects of different media combinations on bud growth, and to analyze the growth differences between treatments.

#### Assessment of culture media for rooting induction

This experiment used 0.5x MS as the base medium and selected 2–3 cm tall plantlets that were robust and well-established through pre-cultivation as the experimental material. The plantlets were inoculated onto rooting induction media containing different concentrations of NAA (0.1, 0.5, 1.0 mg/L) and IBA (0.5, 1.0, 2.0 mg/L) (**Table 6**). A two-factor, three-level orthogonal experimental design was employed, with nine treatment groups. Each group contained nine plantlets, with three replications. After 30 days of cultivation, root formation was observed and recorded for each treatment group.

**Table 6:** Combinations of plant growth regulators in root induction media.

Serial number	Hormones	
	NAA(mg/L)	IBA(mg/L)
1	0.1	0.5
2	0.1	1.0
3	0.1	2.0
4	0.5	0.5
5	0.5	1.0
6	0.5	2.0
7	1.0	0.5
8	1.0	1.0
9	1.0	2.0

### Indirect organogenesis pathway

#### Assessment of culture media for callus induction from leaf explants

Young leaves were used as explants and inoculated onto three different basic media: MS, 0.5x MS, and WPM, each supplemented with selected combinations of 6-BA (1.0, 1.5, 2.0, 3.0 mg/L) and NAA (0.1, 0.5 mg/L). Based on preliminary trials and relevant literature, a total of 12 representative treatment groups were established to evaluate their effects on callus induction (Li et al., 2015; Hua, 2015). Each treatment group included 20 explants, with three replications (**Table 7**). After 30 days of cultivation, callus formation was observed, and the extent of browning was evaluated.

**Table 7:** Experimental design for leaf callus induction under different media combinations.

Serial number	Basal media	Hormone combinations for culture media		Inoculated number
		6-BA(mg/L)	NAA(mg/L)	
1	MS	1.0	0.1	60
2		1.0	0.5	60
3		1.5	0.1	60
4		1.5	0.5	60
5		2.0	0.1	60
6		2.0	0.5	60
7		3.0	0.5	60
8	0.5x MS	1.0	0.5	60
9		1.5	0.5	60
10		2.0	0.5	60
11		3.0	0.5	60
12	WPM	2	0.5	60

The callus induction rate was determined by observing whether white, transparent, or pale yellow-green callus tissue formed at the cut edges of the leaves. The degree of browning was evaluated using a 0–5 scale, where: 0 represents no browning, 1–2 indicates mild browning (browning area <30%), 3 indicates moderate browning (30%-50%), and 4–5 represents severe browning (>50%). Explants that scored 3 or higher were considered to exhibit “significant browning.” The callus induction rate and browning rate for each treatment were then calculated to assess the effect of different plant growth regulator combinations on callus induction and browning of the leaves.

#### Assessment of culture media for callus proliferation from leaf explants

Leaf calli obtained from the primary culture was used as the material and inoculated onto media containing four different combinations of plant growth regulators (**Table 8**) to evaluate their effects on callus proliferation. Each treatment group received 3–4 calli clumps, with three replications. The cultures were maintained under the same conditions for 30 days, with regular observations made to record the growth status, texture changes, and proliferative capacity of the callus in each treatment group. This allowed for the comparison of the effects of different plant growth regulator combinations on callus proliferation.

**Table 8:** Hormone combinations in leaf callus tissue proliferation media.

Serial number	Hormone combinations for culture media
1	0.5x MS+6-BA 2.0 mg/L+NAA 0.1mg/L
2	0.5x MS+6-BA 2.0 mg/L+NAA 0.5mg/L
3	0.5x MS+6-BA 2.0 mg/L+NAA 1.0mg/L
4	MS+6-BA 2.0 mg/L+NAA 0.5mg/L

#### Assessment of culture media for callus differentiation from leaf explants

Well-developed, non-browned callus obtained during the proliferation phase was used as material and inoculated onto media containing different combinations of plant growth regulators (**Table 9**) to evaluate its differentiation capacity. Each treatment group received 5 uniformly sized callus clumps, with three replications. The cultures were maintained under standardized conditions for one month, during which differentiation was regularly observed.

**Table 9:** Hormone combinations for leaf callus tissue differentiation media.

Serial number	Hormone combinations for culture media
1	MS+6-BA 0.2mg/L
2	MS+6-BA 0.5mg/L+IBA 0.2mg/L
3	MS+6-BA 0.2mg/L+NAA 1.0mg/L+IBA 0.2 mg/L
4	MS+6-BA 0.2mg/L+NAA 2.0mg/L+IBA 0.5 mg/L
5	MS+6-BA 0.2mg/L+NAA 2.0mg/L+IBA 0.5 mg/L+AC 0.5 mg/L
6	MS+6-BA 2.0mg/L+NAA 0.5mg/L+KT 1.0mg/L
7	MS+NAA 2.0mg/L+KT 1.0mg/L
8	0.5x MS+NAA 2.0mg/L+KT 1.0mg/L
9	0.5x MS+6-BA 2.0mg/L+NAA 0.5mg/L +KT 1mg/L

Differentiation was assessed through stereomicroscopic observation of the formation of bud primordia. The presence of distinct bud primordia was used as the criterion for determining whether differentiation had occurred. Each treatment group was observed biweekly until adventitious buds were formed. To enhance the accuracy of differentiation assessment, the experiment included multiple independent observations for each treatment group, recording the number of bud primordia at each observation stage and their developmental progress. In the later stages of culture, the number and growth rate of bud primordia were compared to quantify the degree of differentiation for each treatment.

#### Data processing

The data obtained from the experiment were processed by Microsoft Excel and SPSS 26.0. One-way ANOVA and Duncan’s multiple comparisons were also applied(*P*<0.05).

Bud multiplication rate = total number of induced buds/number of inoculated sprouting buds × 100%;

Contamination rate = number of contaminated explants/number of inoculated explants × 100%;

Browning rate = number of browned explants/number of inoculated explants × 100%;

Induction rate = number of explants induced to produce buds/number of inoculated explants × 100%;

callus induction rate = number of explants producing callus/number of inoculated explants × 100%;

## Results

### Effect of different sterilization methods, explant types on sterilization efficacy

Sterilization is essential in tissue culture to reduce contamination. This study evaluated the effects of different sterilization methods and explant types on sterilization efficacy. Sterilization of semi-lignified stem segments with buds collected in the summer of 2023 was first attempted using 2% NaClO for varying durations (3–15 min). The results indicated that even with a prolonged treatment time of 13 minutes, the minimum contamination rate remained as high as 75%, and the overall sterilization effect was unsatisfactory (**Table 10**). Subsequently, 0.1% HgCl_2_ was applied for 12, 13, and 14 minutes to improve sterilization efficiency and reduce both contamination and browning. Following this treatment, contamination rates within 7 days ranged from 55.56% to 66.67%, and the highest browning rate was 12.5%, showing a notable improvement over NaClO. However, contamination commonly occurred within the first two days post-inoculation, mainly manifesting as bacterial growth at the basal ends of the segments or fungal infection around the buds. Additionally, bud sprouting remained limited. Taking both sterilization efficiency and regenerative performance into account, semi-lignified stem segments with buds were excluded from subsequent experiments.

**Table 10:** Effect of sterilization method, type of explants and season of collection on the effectiveness of sterilization.

Harvesting season	Sterilizing reagent	Sterilization time (min)	Type of explants	Inoculated number	Contamination number	Browning number	Contamination rate (%)	Browning rate (%)
Summer	2% NaClO	3	Semi-lignified stem segments with buds	9	9	0	100	0
		5		8	8	2	100	25
		7		11	11	0	100	0
		10		16	14	0	87.5	0
		13		12	9	0	75	0
		15		8	7	3	87.5	37.5
	0.1% HgCl_2_	7	Young leaves	30	1	4	3.33	7.27
			Sprouting buds	15	1	0	6.67	0
			Young stem with buds	15	2	1	13.33	11.11
		12	Semi-lignified stem segments with buds	15	10	1	66.67	6.67
		13		22	13	2	59.09	9.09
		14		18	10	1	55.56	12.5
Spring	0.1% HgCl_2_	4	Young leaves	20	1	5	5	25
			Sprouting buds	25	2	8	8	32
		5.5	Young leaves	20	1	9	5	45
			Sprouting buds	24	1	8	4.17	33.33
		7	Young leaves	20	0	17	0	85
			Sprouting buds	36	0	28	0	77.78

Based on the preliminary findings and referring to the sterilization protocol for “Haierlian” (Fang et al., 2012), this study further tested newly developed young leaves, sprouting buds, and young stem segments with buds as explants, using 0.1% HgCl_2_ for 7nbsp;minutes. The results showed a marked reduction in contamination rates to 3.33%, 6.67%, and 13.33%, respectively, with browning rates maintained between 7.27% and 11.11%. All three explant types exhibited normal initiation, indicating a significantly better sterilization outcome compared to the previously tested material.

In spring 2024, the same explants—young leaves, sprouting buds, and young stem segments with buds—were tested using the optimized 0.1% HgCl_2_ treatment for 7 minutes, as established during the summer experiment. While young leaves and sprouting buds showed extensive browning within five days post-inoculation—characterized by leaf discoloration and necrosis of bud centers—the young stem segments with buds exhibited normal growth and low contamination. For young leaves, both 4-minute and 5.5-minute treatments achieved a contamination rate of 5%; however, the 5.5-minute treatment caused a higher browning rate (45%), making 4 minutes more suitable. For sprouting buds, the 5.5-minute treatment yielded the lowest contamination rate (4.71%) and a browning rate similar to that of the 4-minute treatment, thus considered optimal.

In summary, after optimizing sterilization protocols for each explant type, newly developed young leaves, sprouting buds, and young stem segments with buds demonstrated relatively low contamination and browning rates, suggesting they are suitable explants for the tissue culture of *I. henryi*. Among the sterilization reagents tested, 0.1% HgCl_2_ proved most effective. Under summer collection conditions, a 7-minute treatment effectively controlled contamination and browning. In contrast, for spring-collected materials, the optimal treatment durations were 4 minutes for young leaves and 5.5 minutes for sprouting buds.

### Direct organogenesis pathway

#### Effects of different basal culture media on the induction of sprouting buds

**Figure 2:**
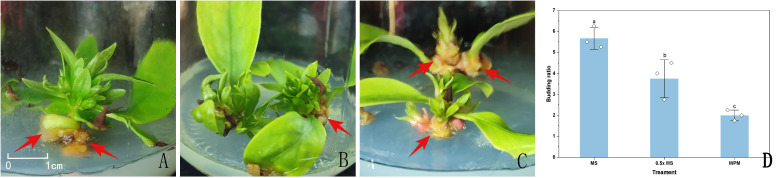
Sprouting buds in different media approximately 20 days after inoculation. **(A)** sprouting buds in MS medium; **(B)** sprouting buds in 0.5x MS medium; **(C)** sprouting buds in WPM medium; **(D)** Effect of different culture media on the induction of sprouting buds. Red arrowheads indicate callus formation observed at the base of the explants. Data in the **(D)** are presented as the mean ± standard deviation (SD) of three independent replicates. Different lowercase letters indicate significant differences between treatments (*P* < 0.05).

Sterilized sprouting buds were inoculated on MS, 0.5x MS, and WPM media, each supplemented with 6-BA (2.0 mg/L) and NAA (0.5 mg/L) (**Table 3**), to assess their bud induction performance. Between 7 and 10 days after inoculation, the leaf primordia of sprouting buds began to unfold. By approximately 20 days, the outer leaves were fully expanded, and the apical buds gradually emerged and elongated, forming multiple axillary buds. At 30 days, the buds had reached a length of 1–2 cm (**Figure 2**).

On MS and 0.5x MS media, the regenerated shoots exhibited vigorous growth, with green, broad leaves and overall healthy morphology. Among the three media, MS showed the highest bud multiplication rate, reaching 5.67 (**Figure 2D**). Shoots on MS medium elongated more rapidly, with clearly swollen apical buds, abundant newly formed leaves, and well-developed, dark green, and flattened leaf blades, indicating strong growth potential. The 0.5x MS medium followed, showing good induction capacity though slightly lower than that of MS. In contrast, shoots induced on WPM medium often displayed red pigmentation at the basal region, pale and narrow leaves, and generally weaker growth vigor. The lowest bud multiplication rate was observed on WPM, and the buds frequently developed abnormally during subculture, with slower growth (**Figure 2**).

Additionally, by approximately 20 days of culture across all media, pale yellow callus formation was occasionally observed at the base of sprouting buds or around the petiole region in contact with the medium surface. The time to initial bud emergence was 25 days on MS medium, 28 days on 0.5x MS medium, and 22 days on WPM medium.

In summary, MS medium not only achieved the highest bud multiplication rate but also induced robust shoot development within a shorter period, indicating superior overall performance in bud induction.

### Effects of different plant growth regulator ratios on the induction of budded stem segments

To investigate the effects of different combinations of plant growth regulators on bud induction from stem segments with buds, an orthogonal experiment with two factors and three levels was designed using 6-BA (1.0, 1.5, and 2.0 mg/L) and NAA (0.05, 0.1, and 0.5 mg/L), resulting in a total of nine treatments (**Table 4**). The results showed that all treatments were capable of inducing bud development. Bud primordia began to swell approximately 10 days after inoculation, and leaves gradually expanded by around 15 days, with bud elongation progressing slowly thereafter.

**Figure 3:**
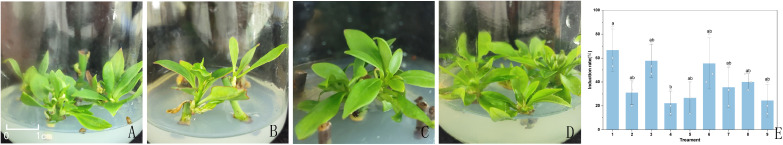
Buds induced in three hormone combinations. **(A)** buds induced by treatment 1; **(B)** buds induced by treatment 3; **(C)** buds induced by treatment 6. **(D)** buds induced by treatment 4. **(E)** Effect of different hormone ratios on the induction of sprouted stem segments. Treatments 1, 3, and 6 were selected as representative results due to their highest induction rates, while treatment 4, with the lowest induction rate, was included for comparison. Data in the **(E)** are presented as the mean ± standard deviation (SD) of three independent replicates. Different lowercase letters indicate significant differences between treatments (*P* < 0.05).

Analysis of the bud induction performance revealed that when NAA was applied at either 0.05 mg/L (treatments 1, 4, and 7) or 0.5 mg/L (treatments 3, 6, and 9), the bud induction rate declined as the concentration of 6-BA increased (**Figures 3B, C**). This suggests that under these NAA levels, lower concentrations of 6-BA were more favorable for initial bud induction and early development. According to **Figure 3E**, treatment 1 (6-BA 1.0 mg/L + NAA 0.05 mg/L) achieved the highest induction rate of 66.67%, with visibly elongated buds, more unfolded leaves, and well-formed morphology, indicating strong growth potential (**Figure 3A**). In contrast, treatment 4 resulted in a much lower induction rate of approximately 22%, with limited bud elongation and small, slowly unfolding leaves, reflecting weaker growth performance (**Figure 3D**).

In conclusion, the combination of MS medium supplemented with 6-BA (1.0 mg/L) and NAA (0.05 mg/L) was the most effective for bud induction from *I. henryi* stem segments with buds. This treatment not only ensured a high induction rate but also supported normal bud development and robust growth. Notably, all explants that successfully induced bud formation contained either apical or axillary buds, suggesting that the exogenous hormone combination primarily promoted the activation and growth of existing meristems rather than inducing dedifferentiation or *de novo* meristem formation.

### Effects of different combinations of plant growth regulators on healthy plantlet development

To promote healthy plantlet development, weakly growing buds derived from bud primordia and axillary buds were individually excised and inoculated onto nine different plantlet-strengthening media formulations (**Table 5**) for a culture period of two months. As all materials were maintained under aseptic conditions, direct measurements such as plant height, stem diameter, and leaf length were not feasible. Therefore, morphological assessment was employed to comprehensively compare the growth performance across treatments.

**Figure 4:**
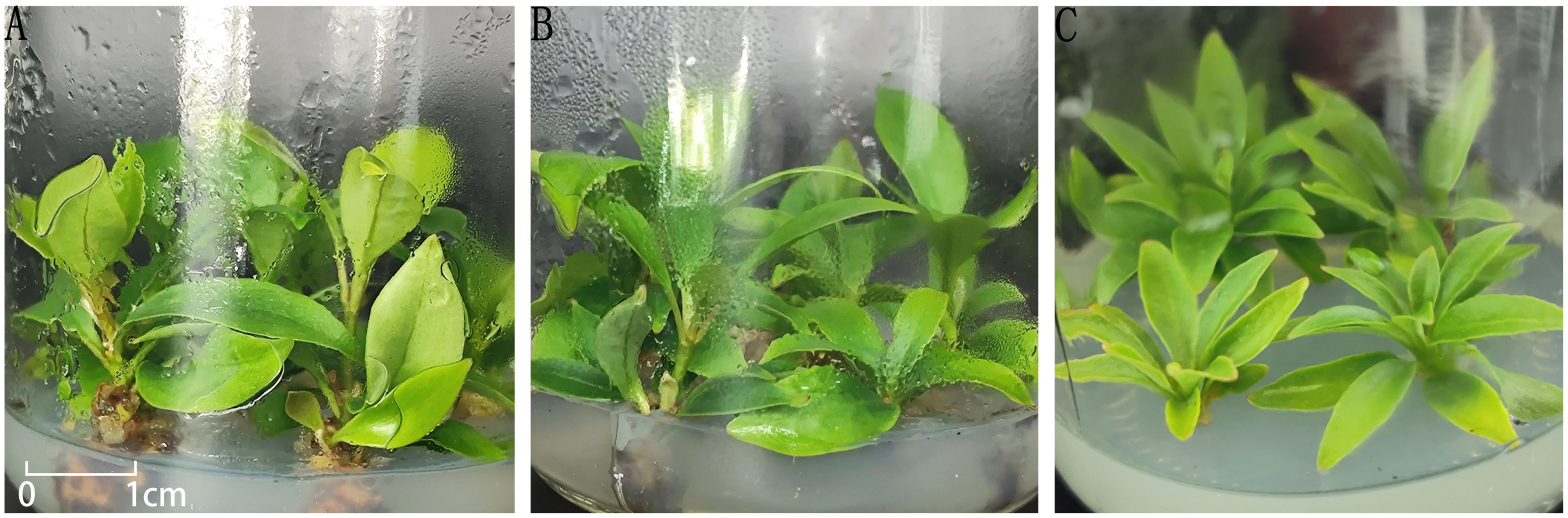
Growth of buds in different treatments. **(A)** plantlets in treatment No. 5; **(B)** plantlets in treatment No. 7; **(C)** plantlets in treatment No. 9.

In treatments 1-4, although the leaf blades of the buds were able to expand and the central buds continued to grow, overall development remained slow. The stems exhibited minimal elongation, and the leaves were generally narrow and small. In contrast, treatment 5 (MS + 6-BA 2.0 mg/L + NAA 0.5 mg/L) showed markedly enhanced bud vigor, with broad, dark green leaves, thickened and elongated stems, and the emergence of new axillary buds, indicating a strong proliferation potential (**Figure 4A**). Treatments 6–8 showed some degree of stem elongation, but overall growth was weaker compared to treatment 5, with thinner stems and less vigorous development (**Figure 4B**). In treatment 9 (MS medium without plant growth regulators), minimal bud growth was observed, with pale green, narrow leaves and weak overall performance (**Figure 4C**).

Taken together, among the nine treatments tested, treatment 5 exhibited the most favorable effect on promoting robust bud growth and is recommended as the optimal formulation for plantlet strengthening in *I. henryi* tissue culture.

### Effects of different combinations of plant growth regulators on root induction

**Figure 5:**
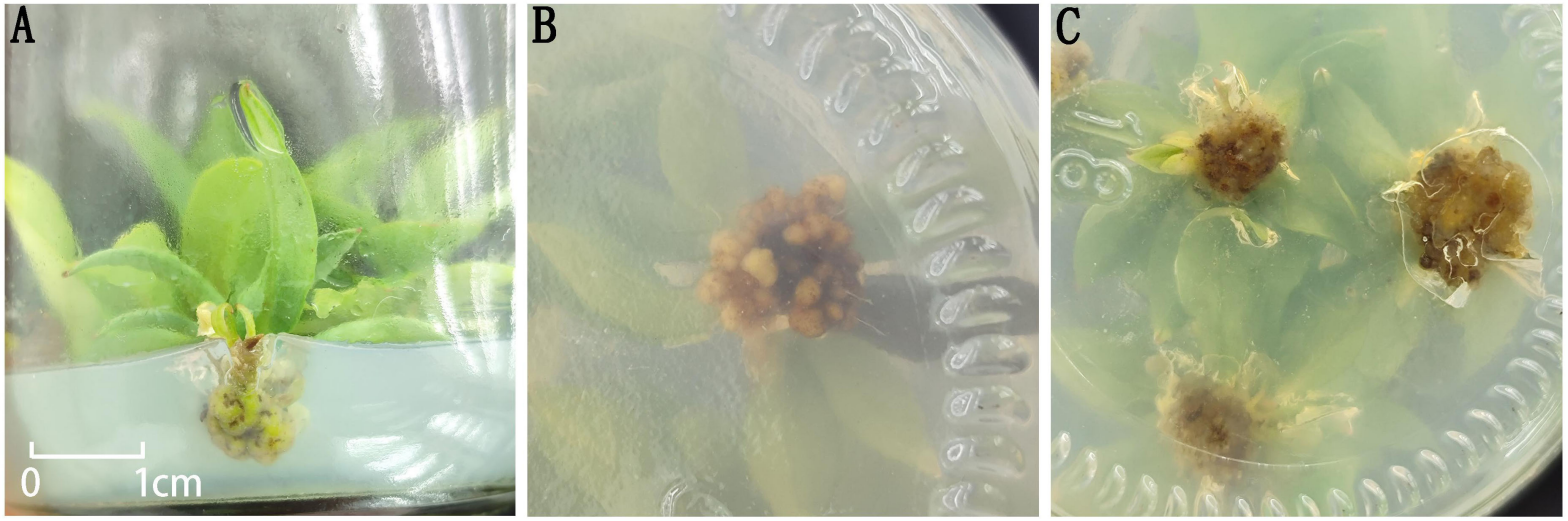
Morphology of callus tissue at the base of plantlets. **(A)** Green callus tissue at the base in Treatment 1; **(B)** Nodular callus tissue at the base in Treatment 1; **(C)** Morphology of callus tissue at the base in the remaining treatments.

After transferring healthy plantlets obtained from the plantlet-strengthening phase to different rooting media (**Table 6**), no significant morphological changes were observed at the base of the plantlets within the first 7 days. By day 15, slight swelling began to appear at the basal cut surface of some plantlets. By day 20, most treatments showed the formation of white, soft callus tissue at the base, whereas only treatment 1 produced greenish, compact callus tissue (**Figure 5**). After one month of culture, the callus in all treatments generally turned pale yellow, except for treatment 1, in which the callus remained compact and exhibited a nodular appearance. However, after one round of subculture, no adventitious root formation was observed in any of the treatments.

Moreover, the composition of plant growth regulators had a significant effect on callus development. When NAA concentration was kept constant, increasing IBA levels promoted a marked increase in the size of callus at the plantlet base. Similarly, under a fixed IBA concentration, higher levels of NAA also enhanced callus proliferation. These results suggest a synergistic effect between IBA and NAA in stimulating callus formation. Nevertheless, under the current experimental conditions, this interaction was still insufficient to induce adventitious root differentiation in *I. henryi* plantlets.

### Indirect organogenesis pathway

#### Effects of different medium combinations on callus induction from leaf explants

**Figure 6:**
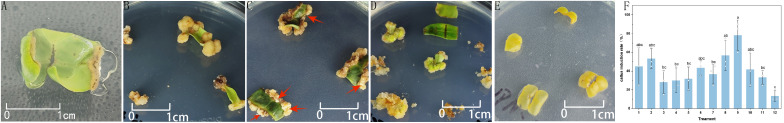
Leaf callus tissues in different induction media. **(A)** yellowish callus in MS medium; **(B)** yellowish callus in 0.5x MS medium; **(C)** white callus in 0.5x MS medium; **(D)** green callus in 0.5x MS medium; **(E)** no callus in WPM medium. **(F)** Effect of different media combinations on the induction of leaf callus. In **(C)**, red arrowheads indicate the relatively whitish areas within the predominantly white-to-brownish callus. Data in the **(F)** are presented as the mean ± standard deviation (SD) of three independent replicates. Different lowercase letters indicate significant differences between treatments (*P* < 0.05).

Following surface sterilization and removal of the petiole and leaf apex, leaf explants were inoculated onto different callus induction media. Across all treatments, varying degrees of swelling appeared on the leaf surface within 5–7 days. By around day 15, slight yellowing was observed at the cut edges, and callus formation was evident at the incision sites after approximately 25 days (**Figure 6**).

Notable differences were observed in leaf coloration and callus morphology among the different media. In MS medium, leaves maintained a vibrant green color, and the induced callus appeared pale yellow and relatively soft in texture (**Figure 6A**). In 0.5x MS medium, the leaves appeared darker in color. The callus was mostly pale yellow or white, with some parts turning green; the texture was denser or appeared as compact clusters (**Figure 6B**). In contrast, leaf explants cultured on WPM medium exhibited chlorosis and yellowing after swelling, with a much lower callus induction rate and poorly developed callus (**Figure 6C**). WPM medium yielded the lowest callus induction rate (13.33%) and was thus deemed unsuitable for further experiments (**Table 7**).

Subsequent optimization focused only on MS and 0.5x MS media by adjusting the concentrations of plant growth regulators. In MS medium (treatments 1–7 in **Table 7**), there were no statistically significant differences (*P* > 0.05) in callus induction rates among different hormone combinations. However, treatment 2 (6-BA 1.0 mg/L + NAA 0.5 mg/L) produced the highest induction rate at 53.33%. In 0.5× MS medium (treatments 8–11), when the NAA concentration was fixed, treatment 9 (6-BA 1.5 mg/L) yielded the highest callus induction rate at 78.33%, which was significantly higher than the other treatments (*P* < 0.05). However, further increasing 6-BA to 2.0 mg/L (treatments 10 and 11) resulted in a decline in induction rates (**Figure 6F**).

A comparison of the three media under the same hormone combination (6-BA 2.0 mg/L + NAA 0.5 mg/L) revealed that 0.5x MS medium performed better than MS, while WPM showed the poorest response, confirming that WPM is not suitable for callus induction from *I. henryi* leaf explants. Overall, the optimal combination for inducing high-quality callus was 0.5x MS medium supplemented with 6-BA (1.5 mg/L) and NAA (0.5 mg/L), which significantly improved both the induction rate and callus quality.

#### Effects of different combinations of plant growth regulators on leaf callus proliferation

**Figure 7:**
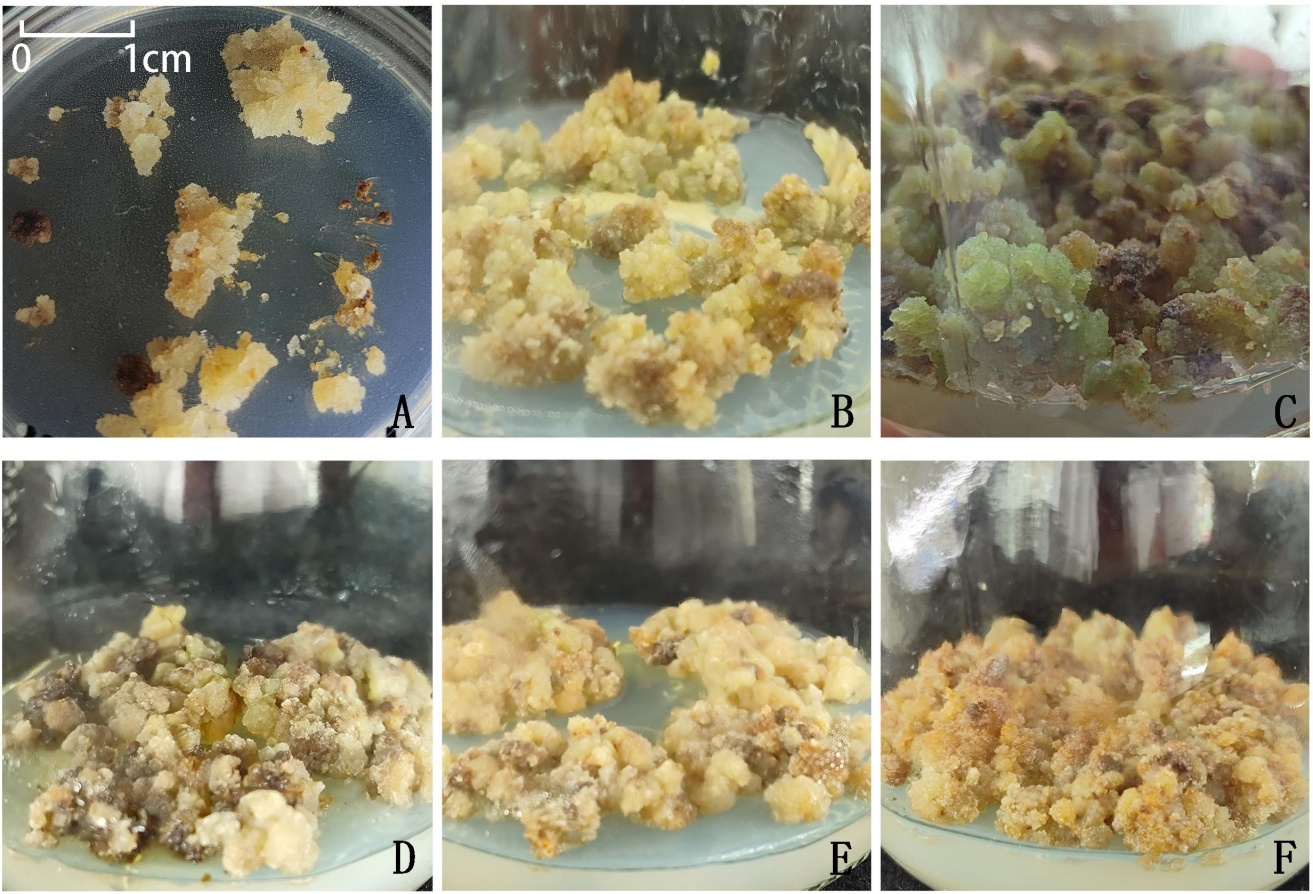
Growth performance of callus tissues on different proliferation media across subculture stages. **(A)** callus tissue obtained from initial induction; **(B)** No. 1 treatment, subcultured once; **(C)** No. 1 treatment, subcultured twice; **(D)** No. 1 treatment, subcultured three times; **(E)** No. 2 treatment, subcultured twice; **(F)** No. 3 treatment, subcultured twice. Each subculture cycle lasted 30 days.

Calli induced from leaf explants were transferred to four different proliferation media for the first subculture (**Table 8**). The results showed that only calli on MS medium (Treatment 4) exhibited severe browning and gradually became necrotic; therefore, this combination was excluded from subsequent experiments. In contrast, calli on the other three combinations, all based on 0.5x MS medium (Treatments 1, 2, and 3), remained pale yellow and increased in size over time. When these calli were subcultured once using the same formulations, those in Treatment 1 gradually turned green in some portions and maintained good tissue status, whereas calli in Treatments 2 and 3 showed no obvious color change and remained pale yellow, with partial browning observed in some tissues. During the third subculture, the amount of green callus in Treatment 1 did not further increase; instead, partial browning occurred in some tissues (**Figure 7**).

#### Effects of different combinations of plant growth regulators on leaf callus differentiation

Among the nine treatments tested for callus differentiation (**Table 9**), a noticeable increase in callus volume was observed only in Treatment 4, where the tissue remained yellow throughout the culture period. However, no adventitious shoot differentiation was detected. Treatment 5, in which 0.5 g/L of activated charcoal was additionally supplemented, also showed no significant promotive effect on callus differentiation. In contrast, calli in the remaining treatments (Treatments 1–3 and 6-9) exhibited varying degrees of browning, eventually turning black and necrotic. Overall, none of the tested conditions successfully induced adventitious shoot formation, indicating that, under the current combinations of plant growth regulators and culture conditions, calli derived from *I. henryi* leaf explants had limited organogenic capacity. The low physiological activity of the calli, coupled with severe browning observed in several treatments, may have constrained their differentiation potential.

## Discussion

### Effects of sterilization methods, explant types, and sampling seasons on sterilization efficiency

in plant tissue culture research, the sterilization of explants is fundamental to the success of subsequent culture processes. The choice of sterilizing agents and their exposure durations plays a decisive role in disinfection efficiency (Jain and Gupta, 2005), while the selection of appropriate explant types is also critical for the establishment of regeneration systems in woody plants (Rachmawati et al., 2024). Due to the low fruit-setting rate of *I. henryi* in its introduced habitat, seeds cannot be reliably used as experimental materials. Therefore, this study employed four types of tissues as explants: current-year semi-lignified stem segments with buds, young leaves, sprouting buds, and tender stem segments with buds. Their responses under various sterilization treatments and across different seasons were systematically evaluated.

The results indicated that 0.1% HgCl_2_ demonstrated superior sterilization efficacy. When young tissues were treated with 0.1% HgCl_2_ for 7 minutes, a low contamination rate was observed, and the explants were able to initiate culture normally. This suggests that younger tissues have a higher success rate for sterilization (Isah, 2016, 2023). Therefore, it is recommended that tender explants treated with 0.1% HgCl_2_ be prioritized for tissue culture of *I. henryi*.

Seasonal variation is also a significant factor affecting the regenerative capacity of woody plant explants (Jayusman and Dalimunthe, 2022). Sampling was carried out irregularly between July 2023 and May 2024, during which it was found that *I. henryi* entered a secondary growth phase in August 2023. Tissues collected during this period exhibited excellent performance in both bud and callus induction experiments. This phenomenon is consistent with previous studies on *Mytilaria laosensis* Lecomte, a related evergreen broadleaf tree species, which also highlighted the advantages of collecting materials during the secondary growth phase (Sun et al., 2024). In contrast, when young tissues were collected in the spring of 2024 and subjected to the same sterilization protocol, only the tender stem segments showed comparable sterilization performance. For sprouting buds and young leaves, shorter exposure times were required to minimize browning. This may be due to the fact that, during early spring, plant cells are metabolically active but have thinner cell walls, making them more vulnerable to damage by disinfectants. In summer, tissues are more mature and exhibit stronger stress resistance, leading to better sterilization outcomes. Prior research has also reported significant physiological differences between spring and summer in terms of dormancy status and metabolic rates (Velappan et al., 2022). Furthermore, seasonal changes in environmental factors such as temperature, light intensity, and day length can alter plant physiology, indirectly affecting explant sterilization and regeneration outcomes. Thus, while spring is also a suitable sampling season, the increased sensitivity of spring-collected young tissues to sterilization treatments necessitates shorter exposure durations to avoid tissue damage.

Comparative callus induction experiments using young leaves collected in spring and summer showed that summer-collected materials exhibited minimal browning post-sterilization, with rapid and healthy callus development. In contrast, spring-collected samples exhibited a browning rate of at least 25%, even with reduced sterilization time, and callus growth was slow, with a higher tendency to brown during subculture. Therefore, young leaf explants collected in summer are preferable for callus induction experiments.

It should be noted that the seasonal analysis in this study is based on data from a single year, without multi-year replication. Given the interannual variability in climate conditions (e.g., temperature, precipitation, insolation), which may influence plant growth rhythms and physiological status, the superior performance of summer-collected materials observed in this study may be partially coincidental and cannot yet be generalized as a consistent trend.

### Effects of different basal media on sprouting bud induction and leaf callus induction via two organogenesis pathways

The selection of an appropriate basal medium is a critical factor in plant *in vitro* culture, directly influencing explant induction, growth, and development. Commonly used media for woody plants include MS, 0.5x MS, and WPM (Oberschelp and Gonçalves, 2016; Phillips and Garda, 2019). Among these, WPM is a modified version of MS and has demonstrated good performance in the tissue culture of various woody species (Zhang et al., 2024). For example, WPM has been successfully applied in the rapid propagation of bud-bearing stem segments of ‘Deyou No. 6’ *Camellia oleifera* (Li et al., 2024), *in vitro* propagation of *Lagerstroemia indica* ‘Midnight’ stem segments (Ma et al., 2024), and optimization of explant culture systems in *Morus alba* (Lu et al., 2021). However, systematic studies on the application of WPM in the tissue culture of *Illicium* species remain scarce in the literature (Fang et al., 2012; Long et al., 2023).

In this context, the present study used sprouting buds and young leaves of *I. henryi* as explants to compare the effects of MS, 0.5x MS, and WPM basal media on sprouting bud induction and young leaf callus induction. The results showed that both MS and 0.5x MS media could successfully induce bud formation; however, MS medium led to a higher multiplication rate and exhibited greater efficiency in shoot proliferation. This may be attributed to the higher concentrations of inorganic salts and a more balanced nitrogen source in MS medium, which are likely more favorable for maintaining meristematic activity. These findings also suggest that direct organogenesis relies heavily on the presence of pre-existing meristematic structures within the explants. This finding is consistent with previous reports highlighting the superiority of MS medium in bud induction (Li et al., 2015). Additionally, during sprouting bud induction, pale yellow callus was observed at the base of the buds and at the junction of the petiole in contact with the medium across all three media types. This suggests that these regions also possess inductive potential. The observation implies that both sprouting buds and their petioles may contribute to regeneration system development in *I. henryi*, offering new perspectives for future studies on regeneration mechanisms and callus utilization.In callus induction from young leaves, 0.5x MS medium outperformed MS and WPM, resulting in more significant induction outcomes.

Therefore, the choice of basal medium should be tailored to specific experimental objectives: MS medium is recommended for shoot induction, whereas 0.5x MS medium is more suitable for callus induction from young leaves.

### Comparative analysis of the effects of different media combinations

#### Optimal media combinations for different culture stages of budded stem segments

Plant growth regulators play a critical role in plant regeneration, which has traditionally been explained by the balance between cytokinins and auxins (Skoog and Miller, 1957). This hormonal ratio is generally considered a key determinant of morphogenesis, with higher cytokinin levels favoring shoot induction and lower ratios promoting callus formation. However, recent studies have revealed a more complex regulatory mechanism. Emerging evidence suggests that dynamic changes in endogenous auxin levels, chromatin state, and nutrient balance are crucial factors influencing regenerative potential (Hussain et al., 2021; Ikeuchi et al., 2019). In addition, the exogenous application of BAP may exert its effects primarily by modulating endogenous auxin homeostasis rather than by directly enhancing cytokinin activity.

In the present study, indirect organogenesis from callus was not achieved. Only explants containing pre-existing meristematic regions, such as sprouting buds or nodal stem segments, were capable of successful regeneration. This indicates that the classical cytokinin-to-auxin ratio model alone cannot fully explain the observed outcomes. Instead, factors such as the initial developmental state of the explant, the presence of active meristems, and potentially epigenetic or hormonal homeostasis appear to be critical for morphogenic competence in *I. henryi*.

### Shoot induction stage

In the present study, semi-lignified stem segments with buds of *I. henryi* were used as explants. The optimal shoot induction medium was identified as MS supplemented with 1.0 mg/L 6-BA and 0.05 mg/L NAA, yielding a cytokinin-to-auxin ratio of 20:1. This ratio differs significantly from previous studies. For instance, in the initial shoot induction of *Illicium* stem segments and *Illicium difengpi* and apical buds, the optimal combination was MS + 1.5 mg/L 6-BA + 0.1 mg/L NAA, with a cytokinin-to-auxin ratio of 15:1 (Li et al., 2015; Long et al., 2023). Similarly, in *Kadsura coccinea*, (Schisandraceae), which belongs to the same family as *Illicium*, the optimal medium was MS + 2.0 mg/L 6-BA + 0.2 mg/L NAA, with a ratio of 10:1 (Deng et al., 2024). These differences may be attributed to the unique physiological characteristics of *I. henryi*, which could result in distinct sensitivity to exogenous PGRs compared with other species. Based on experimental observations, it is speculated that regeneration in *I. henryi* primarily depends on the presence of pre-existing meristematic structures, such as axillary buds or the cambial region of stem segments, rather than occurring through a dedifferentiation-redifferentiation pathway. This regeneration route, classified as direct organogenesis, is likely driven by the activation of existing meristems and therefore may require a lower dependency on the classical cytokinin-to-auxin ratio than systems that rely on *de novo* meristem formation.

### Shoot elongation stage

During the shoot elongation stage, MS medium was used as the basal medium, supplemented with various types and concentrations of PGRs. In the absence of PGRs, plantlets exhibited poor growth. However, the addition of 6-BA in combination with either NAA or IBA significantly improved shoot development. The best result was obtained with MS + 2.0 mg/L 6-BA + 0.05 mg/L NAA, highlighting the essential role of exogenous PGRs in promoting shoot elongation. Furthermore, NAA proved to be more effective than IBA during this stage.

### Rooting stage

Root induction in *I. henryi* plantlets proved to be challenging. In the rooting stage, nine treatments were designed using 0.5x MS as the basal medium, supplemented with different concentrations of NAA (0.1–1.0 mg/L) and IBA (0.5–2.0 mg/L) to induce adventitious roots. However, after two successive subcultures, none of the treatments succeeded in root formation. Instead, as auxin concentrations increased, a noticeable enlargement of the callus at the base of the shoots was observed, with the tissue becoming soft. This suggests that high auxin levels, while promoting callus proliferation, may inhibit further root differentiation. A similar phenomenon has been reported in *Isodon serra* (Li et al., 2023), indicating that further optimization of the concentration and combination of PGRs is still required to achieve effective root induction.

Rooting remains one of the most challenging steps in the *in vitro* culture of woody plants (Yan et al., 2024). Previous studies have shown that low-salt basal media combined with low concentrations of auxins can facilitate rooting, particularly in the absence of cytokinins, where auxin accumulation favors root differentiation (Feito et al., 1996; Zhai and Xu, 2021). In the *Illicium* genus, studies on *Illicium difengpi* shoot rooting demonstrated that 0.5x MS medium supplemented with 1.0 mg/L of NAA, IBA, or IAA could induce rooting, with IAA producing more roots with a more balanced structure, and IBA also being effective, while NAA primarily induced callus formation (Li et al., 2015). Although IAA showed promising results, it was not included in this study. While both NAA and IBA are auxins, their effects can vary significantly depending on the plant species and tissue type. When used alone, their efficacy may be limited, but their combined application at appropriate concentrations may yield synergistic effects (George et al., 2008).

Therefore, future studies should consider expanding the range of PGR types and concentrations, including the introduction of IAA or the combination of all three auxins, to further optimize the adventitious root induction system for *I. henryi*.

### Callus induction from leaf explants at different culture stages

#### Callus induction stage

In the callus induction stage, young leaf explants of *I. henryi* were cultured on 0.5x MS medium supplemented with 1.5 mg/L 6-BA and 0.5 mg/L NAA. Under these conditions, three distinct types of calli were observed: pale yellow friable callus, green compact callus, and white soft callus.

#### Callus proliferation stage

During the proliferation stage, the effects of MS and 0.5x MS media with varying concentrations of PGRs on callus growth were compared. callus cultured on MS medium exhibited significant browning, whereas those on 0.5x MS medium showed reduced browning. Notably, callus on 0.5x MS medium supplemented with 2.0 mg/L 6-BA and 0.1 mg/L NAA transitioned from yellow to green, indicating enhanced viability and differentiation potential. This observation aligns with findings in *Actinidia chinensis* cv. Hongyang, where pale yellow or white friable callus transformed into green, compact callus capable of shoot regeneration when cultured on MS medium with 1.0 mg/L ZT (Shi et al., 2015). However, in the current study, the callus’s green coloration was not stable over time, with browning intensifying during prolonged culture.

The increased browning may be attributed to the high levels of phenolic compounds, such as tannins and lignin precursors, commonly found in woody plants. These compounds are released during cell metabolism, tissue damage, or stress conditions and oxidized by polyphenol oxidases, leading to browning (Rao et al., 2015; Xiao et al., 2017).Previous studies suggest that replacing the culture medium or short-term dark incubation can mitigate browning to some extent (Amente and Chimdessa, 2021). However, browning tends to worsen with successive subcultures, possibly due to changes in callus structure and physiology, reduced stress tolerance, and increased membrane permeability, facilitating phenolic leakage and oxidation (Chen et al., 2014; Zhou et al., 2000).Therefore, the progressive browning observed in this study may result from the combined effects of the unique tissue characteristics of *I. henryi*, medium composition, and subculture strategies.

#### Callus differentiation stage

In the differentiation stage, nine culture conditions were tested for adventitious shoot induction from callus. Despite some pale yellow callus exhibiting changes in color and texture, no adventitious shoots were successfully induced. These results indicate that the failure of shoot regeneration is not solely attributable to an inappropriate cytokinin-to-auxin ratio. The classical view holds that a high cytokinin-to-auxin ratio promotes shoot formation, whereas a lower ratio favors callus formation (Hussain et al., 2021). However, recent studies have emphasized that the regulatory mechanisms underlying organogenesis are far more complex than previously assumed.

Ikeuchi et al. (2019) proposed that organ regeneration is not determined solely by exogenous hormone application, but is instead controlled by a combination of factors, including the dynamic changes in endogenous auxin levels, the chromatin status of cells, and nutrient availability. For example, certain exogenous cytokinins such as BAP may influence regeneration indirectly by modulating endogenous auxin biosynthesis, polar transport, or signaling, rather than simply elevating endogenous cytokinin levels (Hussain et al., 2021). Therefore, the interaction between exogenous and endogenous hormonal pathways likely plays a central role in determining the organogenic route.

In light of the current findings, the failure to induce adventitious shoots from *I. henryi* leaf-derived callus may be attributed to several factors: (1) the chromatin state of the callus cells may lack the plasticity required to initiate organogenic meristem formation; (2) endogenous auxin levels may be insufficient or irregularly distributed, preventing the attainment of the critical threshold necessary for differentiation; and (3) the persistent accumulation of browning compounds may reduce cellular viability and restrict morphogenic potential.

Additionally, callus color can reflect its physiological activity and differentiation potential. Generally, green callus contains chlorophyll and exhibits photosynthetic activity, correlating with higher shoot regeneration capacity (Song et al., 2023). For instance, green callus of *Eucalyptus urophylla* readily forms adventitious shoots (Cai et al., 2014). Conversely, yellowish, friable callus often has lower differentiation potential, as observed in *Spermacoce hispida* (Deepak et al., 2019). However, some studies report that pale yellow callus can still possess high regenerative capacity, such as in *Eucommia ulmoides* (Jin et al., 2014). Besides hormones, environmental factors like light intensity and temperature significantly influence callus differentiation. For example, in *Xanthoceras sorbifolium*, a light intensity of 1300 lx and a temperature of 26°C markedly improved shoot induction rates (Zhou et al., 2025).

It is also important to note that the MS medium used in this study was originally developed for the growth and differentiation of tobacco callus (Murashige and Skoog, 1962). Due to its high salt concentration and specific nutrient composition, MS medium may not be optimal for the organogenic requirements of woody species such as *I. henryi*. Previous research has demonstrated that woody plants often display species-specific and tissue-dependent responses to medium composition and PGRs during indirect organogenesis (Feito et al., 1996; Zhai and Xu, 2021).

Therefore, future studies should take into account the physiological and metabolic characteristics of *I. henryi* callus, including endogenous hormone gradients and epigenetic regulation. A more effective regeneration system may be developed by optimizing the basal medium components, refining hormone combinations, and tailoring environmental parameters to better support the species’ specific organogenic pathway.

## Conclusion

In this study, semi-lignified stem segments with buds, young leaves, sprouting buds, and young bud-bearing stem segments of *I. henryi* were used as explants to systematically investigate the effects of sterilization methods, explant types, basal media, and types of plant growth regulators on tissue culture performance. The main findings are as follows:

(1) Among the sterilization treatments tested, 0.1% HgCl_2_ was identified as the most effective sterilant for *I. henryi* explants. In addition, young leaves collected in summer exhibited better callus induction efficiency than those collected in spring, which may be attributed to their more vigorous physiological state in summer, enhancing their tolerance to sterilization treatment and subsequent culture conditions.
(2) Sprouting buds produced significantly more shoots than bud-bearing stem segments, indicating that they are the most suitable explant type for direct organogenesis. In contrast, leaf explants were more conducive to callus induction, making them more appropriate for indirect regeneration pathways. Direct organogenesis relies on relatively active shoot meristematic tissues, whereas the indirect pathway involves dedifferentiation and subsequent redifferentiation, which are more complex processes influenced by endogenous hormone balance, chromatin status, and culture conditions.
(3) Regarding medium selection, MS medium supplemented with 2.0 mg/L 6-BA and 0.5 mg/L NAA was optimal for shoot induction from sprouting buds; MS medium with 1.0 mg/L 6-BA and 0.05 mg/L NAA yielded the best results for bud-bearing stem segments; and MS medium supplemented with 2.0 mg/L 6-BA and 0.5 mg/L NAA was also optimal for robust plantlet growth. For callus induction from leaf explants, 0.5x MS medium containing 1.5 mg/L 6-BA and 0.5 mg/L NAA was more suitable.

Although this study achieved promising results in bud induction and callus formation, optimal regeneration outcomes were not obtained during adventitious root induction and callus differentiation stages. Therefore, further optimization of plant growth regulator combinations and culture conditions, along with consideration of the dynamic changes in endogenous hormone regulation and cellular differentiation status, is required to establish a more efficient and stable *in vitro* regeneration system for *I. henryi*. This will provide theoretical support and technical foundations for the propagation of its germplasm resources and for tissue culture research on related medicinal plants.

## Data Availability

The original contributions presented in the study are included in the article/supplementary material, further inquiries can be directed to the corresponding author/s.
